# Associations between exposure to workplace bullying and insomnia: a cross-lagged prospective study of causal directions

**DOI:** 10.1007/s00420-020-01618-2

**Published:** 2021-02-06

**Authors:** Morten Birkeland Nielsen, Ståle Pallesen, Ståle Valvatne Einarsen, Anette Harris, Dhaksshaginy Rajalingam, Johannes Gjerstad

**Affiliations:** 1 National Institute of Occupational Health, PB 8149 Dep, 0033 Oslo, Norway; 2 Department of Psychosocial Science, University of Bergen, Bergen, Norway; 3 Norwegian Competence Center for Sleep Disorders, Haukeland University Hospital, Bergen, Norway

**Keywords:** Workplace harassment, Aggression, Sleep, Functioning, Longitudinal

## Abstract

**Objective:**

Workplace bullying has been established as a significant correlate of sleep problems. However, little is known regarding the causal direction between bullying and sleep. The aim of this study was to examine temporal relationships between bullying and symptoms of insomnia.

**Methods:**

Reciprocal and prospective associations between exposure to workplace bullying and symptoms of insomnia were investigated in a national probability sample comprising 1149 Norwegian employees. Data stemmed from a two-wave full panel survey study with a 6-month time interval between the baseline and follow-up assessments. Models with stabilities, forward-, reverse-, and reciprocal associations were tested and compared using Structural Equation Modelling. Analyses were adjusted for age, gender, and the stability in the outcome variables over time. Workplace bullying was assessed with the nine-item Short Negative Acts Questionnaire. Insomnia was assessed with a previously validated three item scale reflecting problems with sleep onset, sleep maintenance, and early morning awakening.

**Results:**

The forward association model, which showed that exposure to workplace bullying prospectively increased levels of insomnia (*b* = 0.08; *p* < 0.001), had best fit with the data [CFI = 0.94; TLI = 0.93; RMSEA = 0.049 (0.046–0.052)]. The reverse association model where insomnia influences risk of being subjected to bullying was not supported.

**Conclusion:**

Workplace bullying is a risk factor for later insomnia. There is a need for further studies on moderating and mediating variables that can explain how and when bullying influence sleep.

## Introduction

Work and sleep are closely interrelated in our everyday life. Most spend the majority of their time as adults either working or sleeping (Mullins et al. 2014). When sleep is poor, workers often attribute this to work-related stressors (Linton et al. 2015). Conversely, poor sleep may subsequently lead to impaired daytime functioning, including reduced work performance (Swanson et al. 2011). Knowledge about how workplace variables and sleep are related is, therefore, highly important, both with regard to how sleep may been improved in workers and how to maintain optimal functioning and performance at the work. Emerging evidence suggests that exposure to workplace bullying may be an highly relevant risk factor for development of sleep problems (Nielsen et al. 2020). Workplace bullying refers to a systematic form of harassment where an employee, persistently and over a period of time, is exposed to negative actions from superiors or coworkers and where the employee finds it difficult to defend him-/herself against these actions due to a real or perceived power imbalance between target and perpetrator (Einarsen 2000).

Even if bullying may be seen as an episodic stressor, due to the longevity of the exposure, workplace bullying can rather be considered as an on-going chronic stressor. Yet, bullying is an escalating process, including both direct (e.g. being openly ridiculed) and indirect forms (e.g. being socially excluded) of harassment, where exposure can vary from occasional acts to full-blown cases of severe victimization (Einarsen 2000), and where even long-term exposure to less frequent, yet systematic, negative acts is related to subsequent detrimental individual outcomes (Hamre et al. 2020). The present study will, therefore, take all levels of exposure into account from the mere occasional, yet ongoing, exposure to bullying behaviors up to the severe cases falling under the more strict definitional criteria of bullying (Einarsen 1999, 2000). In this we will employ the concept of exposure to bullying behaviors which refer to the degree of the exposure as opposed to the concept of “victims of bullying” which focuses on the extreme end of the bullying spectrum (Nielsen et al. 2011).

Compared to other well-established stressors at the workplace such as high job pace, lack of control, conflicting demands, and role ambiguity, and on-going exposure to workplace bullying behaviors represent a direct threat to the personal integrity of those exposed. It is, therefore, not surprising that such bullying has been established as the one of the most prominent work-related predictor of mental distress (Nielsen et al. 2012; Schutte et al. 2014) and sickness absence (Niedhammer et al. 2013). Previous research have also found significant associations between exposure to such bullying and sleep problems (Hansen et al. 2014; Niedhammer et al. 2009; Vedaa et al. 2016). In a recent systematic review and meta-analysis found that targets of bullying had 2.31 higher odds of reporting sleep problems compared to non-bullied workers in cross-sectional studies, while the odds across prospective studies was 1.62 (Nielsen et al. 2020). Furthermore, findings on disturbances in cortisol regulation show that exposure to bullying increases levels of arousal and causes prolonged physiological activation (Hansen et al. 2011), both of which are associated with poor sleep. However, a limitation of the few conducted previous prospective studies is that they have only examined exposure to bullying as a predictor of sleep problems without taking into consideration that sleep problems may also be a risk of subsequent perceived workplace bullying (Nielsen et al. 2020). Consequently, although bullying has been established as a significant correlate and predictor of sleep problems, the bidirectionality between bullying and sleep is still largely unknown.

Even though it has been claimed that reverse causation is unlikely in the relationship between workplace bullying and sleep (Magee et al. 2015), one should be careful dismissing sleep problems as a potential antecedent to perceived bullying without examining this association empirically. According to Nielsen et al. (2020), there are three potential mechanisms suggesting that sleep problems may increase the risk of subsequent victimization of bullying. First, it is possible that employees suffering from sleep problems have a lower threshold for interpreting experiences as negative (Gordon et al. 2017) and that this increase the likelihood for also reporting negative incidences at the workplace as bullying in questionnaire surveys. Second, employees who experience poor sleep, may be more easily frustrated and provoked and, therefore, behave or react in a manner that provoke others, e.g., reacting with frustration and aggression (Kamphuis et al. 2012). This may trigger retaliation in the form of aggression and acts of bullying. Finally, lack of sleep may inhibit an employee’s performance (Litwiller et al. 2017) which again may lead to negative reactions from peers and superiors that may be perceived as, or escalate, into a bullying situation.

As of today, the impact of sleep problems on risk of exposure to workplace bullying has only been examined in three prospective studies. In a study among 1671 Danish employees, Hansen et al. (2014) found that sleep problems, in the form of disturbed sleep, awakening problems, and poor quality of sleep, were associated with an increased risk of subsequent exposure to occasional, but not frequent, bullying 2 years later. In the two other prospective studies, there were no evidence for any relation between bullying and sleep (Johannessen and Sterud 2017; Vedaa et al. 2016). However, an important limitation of these studies is that they examined forward and reverse associations in separate analyses, rather than simultaneously in a reciprocal cross-panel analysis which allows for statistical comparisons of the models and thereby determining the most likely causal direction (Nielsen et al. 2020). Furthermore, most previous prospective studies on the associations between workplace bullying and sleep has utilized relatively long time-lags, often between 2 and 5 years (Nielsen et al. 2020). As the impact of bullying on sleep, and vice versa, conceivably unfold over a relative short time span, there are reasons to question whether the true association will be detected in studies with such long time-lags. By employing a prospective study design with a 6-month time-lag, in a large-scale probability sample of Norwegian employees, the current study will extend previous knowledge on how bullying relates to sleep by providing a simultaneous investigation of forward and reverse relationships between exposure to workplace bullying behaviors and insomnia. Six months was used as a time-lag since this time-frame is considered as a criterion for duration of workplace bullying in definitions of the construct (Einarsen et al. 2011). Furthermore, a time-lag of 6 months is adequate for detecting the accumulated effects that results from chronic and sustained experience of stressors and strain at the workplace, in our case exposure to ongoing bullying behaviors (Ford et al. 2014).

## Methods

### Design and sample

The present study is based on a two-wave survey of sample of the Norwegian working force with a 6-month time-lag between measurement points. A random and representative sample of 5000 employees was drawn from The Norwegian Central Employee Register by Statistics Norway. The Norwegian Central Employee Register is an official register of all Norwegian employees in both private and public organizations, as reported by employers. Sampling criteria were adults between 18 and 60 years of age employed in a Norwegian enterprise. At baseline assessment (T1), questionnaires were distributed through the Norwegian Postal Service during the spring of 2015, with a response rate of 32 percent. Altogether, 1,608 questionnaires were satisfactorily completed and included in the present study. The survey was approved by the Regional Committee for Medical Research Ethics for Eastern Norway (approval 2014/1725). Responses were treated confidentially, and informed consent was provided by the respondents. The second wave of data (T2) was collected 6 months later following the same procedure as the baseline assessment. There were no changes to the survey content. Only participants who responded to the T1 survey was invited to part take at T2. Altogether 1149 respondents participated in the second wave of data collection (72%).

Mean age of the sample was 45.19 (SD = 10.04) years with a range from 21 to 61. The sample consisted of slightly more women (52%) than men (48%). In total, 53.4% were married, 25.8% were common-law partners, 13.7% were unmarried, and 7.1% were widowed, separated, or divorced. With regard to educational level, altogether 9.4% had primary school as highest level, 31.0% had high school, 32.0% had lower level university, while 27.8% had higher-level university or PhD. A total of 89.4% were in full-time employment, 6.6% in part-time employment, and 3.5% were on a sick leave or occupational rehabilitation, whereas 0.5% was disabled pensioners or retired. Respondents that were on sick leave or occupational rehabilitation, or that turned out to be on disability benefits were excluded from the current study. Altogether 36% had a leadership position with personnel responsibilities, indicating an overrepresentation of leaders and managers in the sample. The final prospective sample comprised 1103 respondents.

### Attrition analyses

Analyses of attrition from T1 to T2 showed no significant differences in T1 data between respondents and non-respondents at T2 with regard to levels of exposure to workplace bullying behaviors (*t* = 0.80; *df* = 1586; *p* > 0.05) or insomnia symptoms (*t* = − 0.32; *df* = 1594; *p* > 0.05). With regard to demographic characteristics, T2 respondents (*M* = 46.75; SD = 18.85) were significantly (*t* = 4.57; *df* = 1603; *p* < 0.001) older than non-respondents (*M* = 42.49; SD = 10.45). However, there were no differences with regard to gender distribution (*χ*^2^ = 1.31; *df* = 1; *p* > 0.05), having leadership responsibility (*χ*^2^ = 1.94; *df* = 1; *p* > 0.05) or educational level (*χ*^2^ = 6.48; *df* = 4; *p* > 0.05). With exception of the difference in age, the findings indicated that the T2 sample was representative for the overall sample.

### Instruments

Ongoing exposure to bullying behaviors in the workplace was measured with the nine-item Short Negative Acts Questionnaire-Revised (SNAQ-R) inventory (Einarsen et al. 2009; Notelaers et al. 2018)*.* SNAQ-R describes negative and unwanted behaviors that may be perceived as bullying if occurring on a regular basis. All items are formulated in behavioral terms and hence focus on the mere exposure to inappropriate behaviors while at work, with no references to the term bullying (Einarsen and Nielsen 2015). The SNAQ-R contains items referring to both direct (e.g., openly attacking the victim) and indirect (e.g., social isolation, slander) negative behaviors (Einarsen et al. 2009). The items do also distinguish between personal and work-related forms of bullying (Einarsen et al. 2009). Example items are “Being ignored or excluded”, “Repeated reminders of your errors or mistakes”, and “Someone withholding information which affects your performance”. The SNAQ focuses on exposure over the past 6 months. The respondents were asked to indicate how often they had been exposed to each specific behavior described by the nine items at their present worksite during the past 6 months. Response categories ranged from 1 to 5 (‘never’, ‘now and then’, ‘monthly’, ‘weekly’ and ‘daily’). The nine-item version of the SNAQ-R had a Cronbach’s alpha of 0.86 at T1 and 0.87 at T2 in the present study.

Insomnia was assessed with a validated three-item scale reflecting problems with sleep onset, maintenance of sleep, and early morning awakening (Pallesen et al. 2019). The items are “Difficulties falling asleep”, “Difficulties with having continuous sleep”, and “Too early awakening in the morning”. The items reflect core nocturnal characteristics of insomnia, in line with modern nosology (American Academy of Sleep Medicine 2014; American Psychiatric Association 2013). Response alternatives ranged from 1 to 4 (‘not bothered’, ‘a little bothered’, ‘considerably bothered’, ‘seriously bothered’). The Cronbach’s alpha for the insomnia scale was 0.81 at T1 and 0.80 at T2.

### Statistical analyses

Descriptive analyses and structural equation models (SEM) were performed with STATA 16.0. The maximum likelihood with missing values (MLMV) estimator in Stata was employed to determine model fit and magnitude of relationships. To determine model fit, Chi-squared (*χ*^2^) test, root mean square error of approximation (RMSEA), Tucker–Lewis Index (TLI) and comparative fit index (CFI) were calculated. Values of RMSEA below 0.05 and values of CFI and TLI above 0.95 were considered indicative of a well-fitting model (Hu and Bentler 1999). All study constructs were modeled as latent variables using their respective observed indicators. The error terms of each indicator at T1 were allowed to co-vary with the corresponding indicator at T2. In addition, synchronous correlations between constructs at the same time-point were allowed in all models. The structural regression analyses of reciprocal time-lagged relationships between workplace bullying and insomnia were conducted in four steps with the following models investigated and compared:

1. *Stability model* (M1). This model estimated the magnitude of the stability in exposure to workplace bullying behavior and insomnia, respectively, across the two time points. No cross-lagged relations between the variables were included in this model.
2. *Forward causation model* (M2). This model is similar to M1, but tests the cross-lagged relation from the exposure to workplace bullying behavior at T1 to insomnia at T2.
3. *Reverse causation model* (M3). This model is similar to M1, but tests the cross-lagged relation from insomnia at T1 to exposure to workplace bullying behavior at T2.
4. *Reciprocal causation model* (M4). This model is similar to M1, but include a simultaneous test of the normal and reverse causation relations tested in models M2 and M3. That is, insomnia at T2 is regressed on bullying at T1 while bullying at T2 is regressed on insomnia at T1.

Through specifying and testing a full cross-lagged autoregressive model, it was possible to contrast and determine the causal/bidirectional relationships between exposure to workplace bullying behavior and insomnia. Although existing evidence is inconclusive, studies have shown age differences (De Cuyper et al. 2009) and gender differences (Glambek et al. 2018) in outcomes of workplace bullying. There are also important age (Carrier et al. 1999) and gender (Krishnan and Collop 2006) differences in sleep problems. We, therefore, controlled for age and gender in the analyses. As leaders and managers were overrepresented in the sample, we also adjusted for whether the participants were in leadership positions.

## Results

### Descriptive statistics

**Fig. 1:**
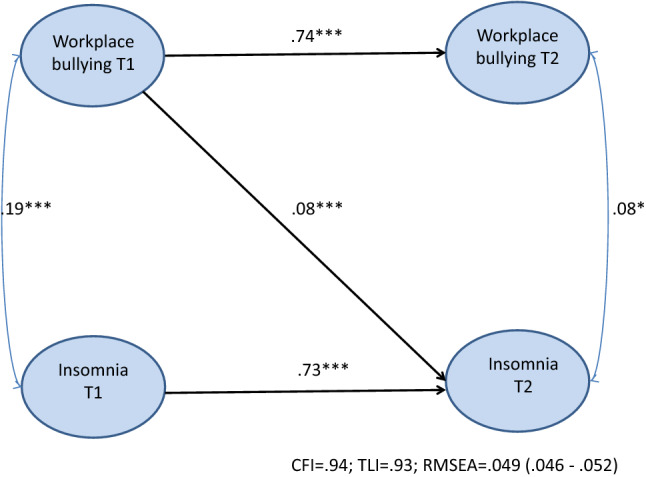
Cross-lagged associations between exposure to workplace bullying and insomnia adjusted for age, gender, and leadership position (M2 model)

Means, standard deviations, and intercorrelations for all study variables are presented in Table 1. Exposure to workplace bullying behavior had a mean score of 1.20 (SD = 0.34) at T1 and 1.19 (SD = 36) at T2. These scores are in line with previous studies using the short Negative Acts Questionnaire (Conway et al. 2018; Leon-Perez et al. 2019; Notelaers et al. 2018). Insomnia had a mean score of 1.69 (SD = 0.75) at T1 and 1.74 (SD = 0.72) at T2. Age was significantly and positively correlated with levels of insomnia at both T1 (*r* = 0.09; *p* < 0.01) and T2 (*r* = 0.07; *p* < 0.05), whereas female gender was associated with higher levels of insomnia at both time points (T1: *r* = 0.06; *p* < 0.05/T2: *r* = 0.07; *p* < 0.05) (Fig. 1). Leadership position was unrelated to exposure to workplace bullying behavior and insomnia at both time-points and across time. Exposure to workplace bullying behavior at T1 correlated positively with levels of insomnia at T1 (*r* = 0.26; *p* < 0.001) and T2 (*r* = 0.26; *p* < 0.001). Insomnia at T1 was significantly and positively associated with exposure to workplace bullying behavior at T2 (*r* = 0.21; *p* < 0.001).

**Table 1:** Descriptive statistics and intercorrelations between study variables (*N* = 1103)

Variable		*M*	SD	1	2	3	4	5	6	7
1	Age	45.52	16.98	–						
2	Gender	0.52	0.50	− 0.03	–					
3	Leadership position	0.36	0.48	0.08**	− 0.20***					
4	Workplace bullying T1	1.20	0.34	− 0.05*	− 0.02	0.01	–			
5	Insomnia T1	1.69	0.75	0.09**	0.06*	− 0.03	0.26***	–		
6	Workplace bullying T2	1.19	0.36	− 0.03	− 0.04	0.05	0.69***	0.21***	–	
7	Insomnia T2	1.74	0.72	0.07*	0.07*	− 0.01	0.26***	0.70***	0.25***	–

*p < 0.05; **p < 0.01; ***p < 0.001

### Measurement model

To assure that workplace bullying and insomnia are empirically different constructs, we followed a confirmatory approach with two distinguishable measurement models. These was a one-dimension model with all items measuring the same latent variable (CMIN = 2020.45.96; *df* = 5; *p* *< *0*.*001*;* CFI = 0*.*73; TLI = 0.67; RMSEA = 0.151; 95% CI RMSEA = 0*.*145–0.156), and a two-dimension model with the workplace bullying items loading on one factor and the insomnia items loading on a separate factor (CMIN = 367.59; *df* = 53; *p* < 0.001; CFI = 0.96; TLI = 0.95; RMSEA = 0.061; 95% CI RMSEA = 0.055–0.067). The fit statistics and comparisons of models indicated that the two-dimensional model had the best fit to data, thus suggesting that exposure to workplace bullying behavior and insomnia represent empirically distinguishable constructs. The correlation between the two latent variables at T1 was 0.26 (*p* < 0.001). This two-factor model had similar fit at T2 (CMIN = 291.88; *df* = 53; *p* < 0.001; CFI = 0.96; TLI = 0.95; RMSEA = 0.063; 95% CI RMSEA = 0*.*056–0.070), with a correlation of 0.23 (*p* < 0.001) between the two latent variables.

### Model comparisons and time-lagged associations

Model comparisons of forward, reverse, and reciprocal relationship between exposure and workplace bullying behavior and insomnia were carried out in order to determine bidirectional relationships between the variables. In the analyses, the different models were tested and compared using the stability model as a reference. The different structural models were compared using chi-square difference tests of nested models. Observed values which were statistically significantly larger than critical values of the chi-square distribution were taken as evidence of support for adding parameters to the model (i.e. the more complex model adds useful information over the more parsimonious one) (Jöreskog 1993). Fit statistics and comparisons are presented in Table 2.

**Table 2:** Results of cross-lagged full panel structural regression between workplace bullying and insomnia

		Test statistics					Model comparisons		
		*χ*^2^	*df*	CFI	TLI	IRMSEA (90% CI)	Comparison	*df*	***χ***^**2**^
M1	Stability model	1095.33***	303	0.94	0.93	0.049 (0.046–0.053)			
M2	Forward model (Workplace bullying T1 → insomnia T2)	1084.50***	302	0.94	0.93	0.049 (0.046–0.052)	M2 vs. M1	10.83**	1
M3	Reverse model (Insomnia T1 → workplace bullying T2)	1095.30***	302	0.94	0.93	0.050 (0.046–0.053)	M3 vs. M1 M3 vs. M2	0.03^NS^ –10.80**	1
M4	Reciprocal model (Workplace bullying T1 → insomnia T2 and insomnia T1 → workplace bullying T2)	1084.48***	301	0.94	0.93	0.049 (0.046–0.052)	M4 vs. M1 M4 vs. M2 M4 vs. M3	10.85** 0.02^NS^ 10.82**	2 1 1

*p < 0.05; **p < 0.01; ***p < 0.001, *NS* = not significant

The stability model (M1) showed acceptable fit to the data [CMIN = 1095.33; *df* = 303; *p* < 0.001; CFI = 0*.*94; TLI = 0.93; RMSEA = 0.049; 90% CI RMSEA = 0*.*046–0.043]. Both exposure to workplace bullying behavior (*b* = 0.74; *p* < 0.001) and insomnia (*b* = 0.75; *p* < 0.001) had high temporal stability over the 6-month time-period. The competing models M2, M3, and M4 were tested against the M1 stability model and against each other. As displayed in Table 3, the M2 forward model showed significantly better fit compared to the M1 stability model. The M3 reverse model did not improve the fit compared to the M1 stability model and had significantly poorer fit compared to the M2 forward model. The M4 reciprocal model had significantly better fit than the M1 stability model and the M3 reverse model, but did not improve the fit when compared to the M2 forward model. This suggests that the M2 forward model provided the best representation of the data. The findings show that exposure to workplace bullying behavior at T1 was associated with a significant increase in insomnia symptoms over time (*b* = 0.08; *p* < 0.001), whereas levels of insomnia at T1 were not associated with any changes in subsequent levels of reported bullying. Neither age, gender, nor leadership was associated with changes in exposure to workplace bullying behavior or insomnia over time.

**Table 3:** Tested associations between indicators of workplace bullying and insomnia in the M2 forward model (standardized coefficients)

Relationship	*B*	95% CI *b*	Standard error
Insomnia T1 → Insomnia T2	0.73***	0.69 to 0.76	0.02
Workplace bullying T1 → Workplace bullying T2	0.74***	0.71 to 0.77	0.02
Workplace bullying → Insomnia T2	0.08***	0.03 to 0.13	0.03
Age → Insomnia T2	0.01	− 0.04 to 0.06	0.02
Gender → Insomnia T2	0.04	− 0.01 to 0.09	0.02
Leadership position → Insomia T2	− 0.02	− 0.07 to 0.02	02
Age → Workplace bullying T2	− 0.02	− 0.06 to 0.03	0.02
Gender → Workplace bullying T2	− 0.02	− 0.07 to 0.03	0.02
Leadership position → Workplace bullying T2	− 0.03	− 0.08 to 0.18	0.02

*p < 0*.*05; **p < 0*.*01; ***p < 0.001

## Discussion

Although exposure to workplace bullying has previously been established as an important correlate of sleep problems, there is a shortage of studies that can shed light on the causal/bidirectional relationship between the variables (Nielsen et al. 2020). Extending previous research, the present study examined bidirectional associations between exposure to bullying behaviors and symptoms of insomnia using a prospective two-wave study design. Comparisons of different causal models showed that a forward causation model, where exposure to workplace bullying behaviors was related to an increase in insomnia over time, had the best fit with the data. The established beta coefficient of 0.08 equals an Odds Ratio of 1.34 (95% CI 1.08–1.66). Hence, the magnitude of the association aligns with the estimate from the meta-analysis on bullying and sleep by Nielsen et al. (2020). We found no evidence for a reverse association where insomnia influenced the risk of being bullied prospectively. The analyses were adjusted for stability in the outcome variable over time, as well as for the impact of age, gender, and leadership position. Taken together, the findings support previous longitudinal evidence for bullying as a risk factor for sleep problems, but provide no evidence for the suggestion that sleep problems are potential precursors for bullying (Nielsen et al. 2020).

The work arena is highly important with regard to both the economic situation, the personal identity, as well as the health and well-being of an employee. It is, therefore, not surprising that being a target of prolonged bullying in the form of harassment and social exclusion at work has severe and detrimental consequences for the sleep of those exposed. Being exposed to bullying typically challenges the world view of those targeted, particularly in early phases where acts tend to be rather subtle and discrete, and may, therefore, easily lead to worries and rumination. This kind of repetitive thoughts about negative work experiences and the inability to switch off from such thoughts (Demsky et al. 2019) have been established as risk factor for sleep problems (Pillai and Drake 2015) and has also been proposed as intervening mechanisms in the relationship between exposure to bullying and sleep (Demsky et al. 2019). The concerns and worries associated with workplace bullying may increase arousal and physical activation which subsequently disturb established sleep patterns (Hansen et al. 2006, 2011). Indeed, a number of studies show that exposure to bullying is associated with symptoms of posttraumatic stress (Nielsen et al. 2015), a form stress response that among others is characterized by hyperarousal, nightmares, and other sleep difficulties (American Psychiatric Association 2013).

There may be several explanations for why we did not find any association between existing symptoms of insomnia and subsequent risk of being exposed to bullying behaviors. First of all, despite theoretical arguments for such a relationship reviewed above, it may be that sleep problems simply do not have any substantial impact on ones exposure to workplace bullying. However, it may also be that an association between sleep problems and workplace bullying is determined by third variables that were not examined in the present study. For instance, it may be that receiving some sort of treatment for insomnia, including use of medication, are beneficial with regard to functioning at the workplace (Kalmbach et al. 2019) and that treatment, therefore, should be assessed as a moderating variable when examining bullying as a consequence of sleep problems. Methodological explanations, such as the length of the time-lag between measurement points, should also be considered. We used a 6-month interval and other findings may have been obtained with longer or shorter time-lags.

Although the aim of the present study was to determine the causal directions between exposure to workplace bullying and sleep, it should be noted that there may be several factors not accounted for in this study that could confound this association. As the causes of sleep problems are complex and multifactorial, determining the impact of other work-related exposures may be important. Previous research has shown that high job pace, lack of control, conflicting demands, role ambiguity and conflict, and social support are especially prominent predictors of both sleep problems (Linton et al. 2015; Vleeshouwers et al. 2015) and workplace bullying (Hauge et al. 2007; Van den Brande et al. 2017). Hence, future studies on the associations between workplace bullying and sleep should, therefore, consider adjusting for these work factors. In addition, since both bullying and sleep problems are related to a range of mental health problems, including anxiety and depression (Hall et al. 2000; Nielsen et al. 2014; Vedaa et al. 2016), future research may also benefit from adjusting for the from such psychological variables.

### Methodological strengths and limitations

In terms of strengths, the present study examined bidirectional relationships between exposure to workplace bullying behavior and sleep in a large and heterogeneous sample using time-lagged full panel data. The sample was drawn from a representative pool of Norwegian employees and can, therefore, be categorized as a probability sample. With exception of being somewhat older, the attrition analyses indicated that the cohort was representative for the overall baseline sample regarding relevant demographic factors and on the study variables. Psychometrically sound measurement instruments were used to assess exposure to workplace bullying and insomnia.

Still, some limitations should be noted. First of all, the baseline response rate of 32% was lower than the average rate established for survey studies (Baruch and Holtom 2008), which may call for questioning the external validity of the findings. However, as response rate has limited impact on the internal validity of a study (Schalm and Kelloway 2001), the response rate in the present study should not challenge the actual findings. There is also a strong secular trend of reductions of response rates, and in that context the response obtained in the present study is not deviating (Stedman et al. 2019).

Because all instruments were based on self-report, the study could be influenced by biases such as response set tendencies and social desirability. In addition, there is also the possibility that the results could have been influenced by the common method variance; however, the use of a time lag between the measurement of the independent and dependent variables in the current study has most likely curbed this risk (Podsakoff et al. 2003).

In the present study, we used a 6 months’ time-lag. This time-lag is adequate for detecting the accumulated effects that result from the chronic and sustained experience of stressors and strain (Ford et al. 2014). However, since the social repercussions of some sleep difficulties may take more than 6 months to develop, it should be noted that this time-lag favors detecting a link between bullying and subsequent sleep problems rather than the reverse. Hence, upcoming studies on the bidirectional associations between workplace bullying and sleep problems should consider using other time-lags.

### Implications and conclusions

The present study has two main take-away messages. First, ongoing exposure to bullying behaviors at the workplace is a risk factor for subsequent insomnia. Second, sleep problems in the form of insomnia seem to have no impact on the risk of subsequent exposure to bullying at the workplace. Hence, the study has addressed one important knowledge gap in research on this topic, namely the directionality of the association between bullying and sleep (Nielsen et al. 2020). However, as the study did not include any information about underlying mechanisms or conditions that can explain how and when bullying leads to insomnia, there is a need for upcoming studies on potentially mediating and moderating variables, preferably using longitudinal research designs (e.g., such as used by Pereira et al. 2013) and objective measures of sleep quantity and quality (Nielsen et al. 2020).

Regarding practical implications, the findings of the present study indicate that measures against bullying at the workplace can be beneficial concerning reducing sleep problems among employees. Previous research has shown that perceived organizational support and an ethical climate are beneficial with respect to reducing the effects of bullying (Einarsen et al. 2018; Nielsen et al. 2019). Such measures may, therefore, also be helpful reducing sleep problems following exposure to bullying. Future research should determine the effectiveness of these kind of measures with regard to sleep.
